# Cutaneous leiomyosarcoma arising in a smallpox scar

**DOI:** 10.1186/1477-7819-10-148

**Published:** 2012-07-16

**Authors:** Robert A Pol, Hilde Dannenberg, Jan-Lukas Robertus, Robert J van Ginkel

**Affiliations:** 1 Department of Surgery, University Medical Center Groningen, P.O. Box 30 001, Groningen, RB, 9700, The Netherlands; 2 Department of Pathology, University Medical Center Groningen, University of Groningen, Groningen, The Netherlands

**Keywords:** Cutaneous leiomyosarcoma, Scar, Small pox, Treatment

## Abstract

**Background:**

Cutaneous leiomyosarcoma (CLM) is a very rare smooth muscle tumour that accounts for about 2–3% of all superficial soft tissue sarcomas. Although the development of various malignancies in scar tissue is well known, we report the first case of a CLM developing in a small pox scar.

**Case presentation:**

A 66-year-old man presented with a painless, slow-growing lump in a small pox scar on his left shoulder. Histological biopsies showed the lesion to be a primary, well-differentiated cutaneous leiomyosarcoma. A CT scan of the thorax was conducted, which showed no signs of metastases. The complete lesion was then surgically excised, and histopathological examination revealed a radically excised cutaneous type leiomyosarcoma After 13 months’ review the patient was doing well with no evidence of tumour recurrence.

**Conclusions:**

This is the first report of a CLM arising in a small pox scar. Although the extended time interval between scarring and malignant changes makes it difficult to advise strict follow-up for patients with small pox scars, one should be aware that atypical changes and/or symptoms occurring in a small pox scar could potentially mean malignant transformation.

## Background

Cutaneous leiomyosarcoma (CLM) is a very rare smooth muscle tumour that accounts for about 2–3% of all superficial soft tissue sarcomas [1]. CLM presents in persons of all ages but with a peak occurrence between 50–70 years of age. It may occur anywhere on the body with a predilection for the limbs. Clinical appearance of CLM is non-specific with a wide range of differential diagnoses including squamous cell carcinoma, amelanotic melanoma and basal cell carcinoma [2]. Although the development of various malignancies in scar tissue is well known, the highest association is found in chronic burn scars. However, sarcomas develop very rarely in (burn) scars. A case of a CLM developing in a small pox scar is presented. To our knowledge, this is the first report of such an association.

## Case presentation

**Figure 1:**
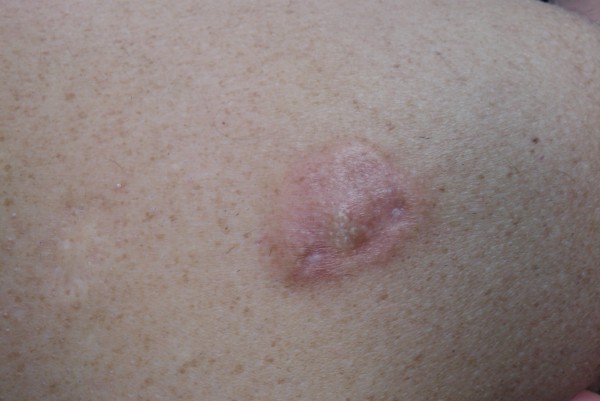
**Macroscopic view of the scar**. The nodular aspect of the small pox scar is clearly visible.

**Figure 2:**
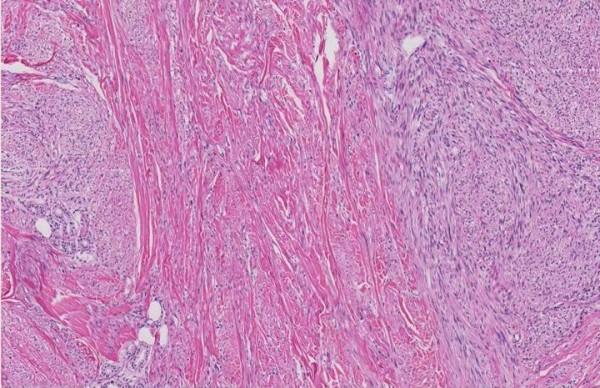
**Histopathological features.** Dermal proliferation of elongated cells arranged in intersecting fascicles [hematoxylin & eosin, original magnifications: (Figure 2) × 5; (Figure 3) × 20].

**Figure 3:**
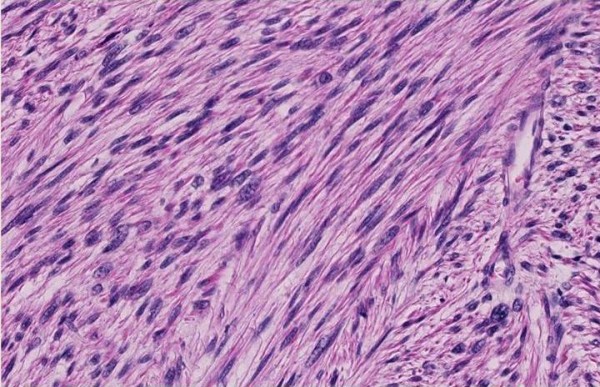
**Histopathological features.** Dermal proliferation of elongated cells arranged in intersecting fascicles [hematoxylin & eosin, original magnifications: (Figure 2) × 5; (Figure 3) × 20].

**Figure 4:**
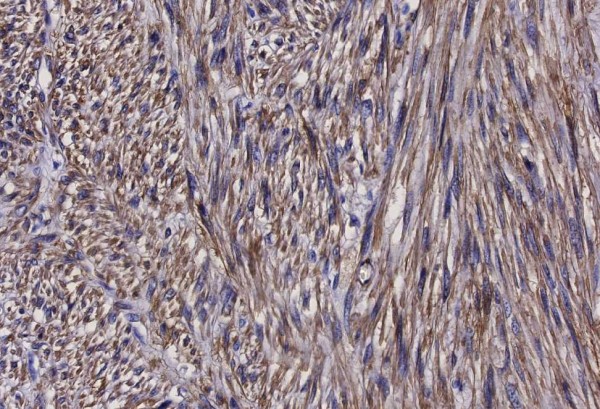
**Immunohistochemical stain for α-smooth muscle actin: (Figure**4**) × 10**.

A 66-year-old man was referred to our outpatient clinic by his dermatologist. He presented with a painless, slow-growing lump in a small pox scar on his left shoulder (Figure 1). Histological biopsies were taken and reviewed by a specialised panel for soft tissue tumours, which showed the lesion to be a primary, well-differentiated cutaneous leiomyosarcoma. On re-examination, a firm, nodular scar was seen with no signs of ulceration. There was no lymphadenopathy in his axilla and/or neck. Additionally, a CT scan of the thorax was conducted, which showed no signs of metastases. The complete lesion was then surgically excised and submitted for further pathological examination (Figures 2 and 3). Histopathological examination revealed a poorly delineated tumour located in the dermis with nodular extension into the superficial subcutis and showing a fasciculated growth pattern of spindle cells with eosinophilic cytoplasm and hyperchromatic, elongated, blunt-ended and pleomorphic nuclei. The mitotic index was less than 10/7 HPF (1.7 mm). Neoplastic cells showed reactivity to α-smooth muscle actin and desmin (Figure 4).

No necrosis was found. Clear surgical margins (> 5 mm) were confirmed histologically. In conclusion, it concerned a radically excised cutaneous type leiomyosarcoma, Coindre type 1. At 13-month review, the patient was doing well with no evidence of tumour recurrence. A control chest x-ray after 6 and 12 months showed no evidence of metastases.

## Discussion

CLM are very rare tumours. They typically present with skin changes such as discolouration and ulceration, and are generally small at presentation (1–2 cm). Due to their low incidence and atypical presentation, they are often misdiagnosed. The most effective treatment of CLM is wide excision with a 3-5-cm lateral margin and a depth that includes the subcutaneous tissue and fascia [3,4]. Local excision without adequate margins leads to recurrence, and increases the risk for metastatic and possibly fatal disease.

Leiomyosarcoma of the skin can be classified as either superficial (cutaneous or subcutaneous) or metastatic leiomyosarcoma from a distant visceral site such as the uterus or retroperitoneum [2]. Differentiation between the cutaneous and subcutaneous subtypes is not always straightforward, and the distinction is made on the basis of different histological features and biological behaviour [5]. As in our patient, CLM occurs most often on the extremities [6]. The metastatic potential differs significantly between the histological subtypes, with the cutaneous variant having a very low rate of distant metastasis and a local recurrence rate of 30% [3]. In this specific case, it also concerned a CLM and, because of the clear surgical margins, has an excellent prognosis.

The aetiology of these tumours is relatively unknown, although antecedent traumatic injury, ionising irradiation, chemicals, sunlight and lupus vulgaris have been associated with this type of tumour [7]. We here describe the first patient with CLM arising in a small pox scar. There are various reports on CLM with a similar association with scars [7-9]. The most common association, however, is found in burn scars. The pathogenesis of malignant transformation in burn scars is not known, mainly because of the low incidence with only 11 cases reported in the literature [9].

Besides the occasional occurrence of CLM, a familial occurrence of cutaneous leiomyosarcoma with renal cancer has been described in the context of hereditary cutaneous leiomyomatosis and renal cell cancer (HLRCC) [10,11]. This rare inherited tumour syndrome is caused by germ line mutations in the fumarate hydratase (FH) gene [12]. However, mutations in FH do not explain sporadic CLM formation in scar tissue, since aberrant FH expression or somatic mutations are not seen in sporadic tumours [13,14]. Adjuvant therapies include radiation therapy, chemotherapy and super voltage cobalt therapy, although CLM has been reported to be both radio- and chemotherapy resistant. [15]

Because this is the first report of a CLM arising in a small pox scar, the presence of a common pathway to malignant transformation with burn scars cannot be determined. Although only a few risk factors have been identified, the most obvious relation with scar tissue has to be the antecedent traumatic injury. The extended time interval between scarring and malignant changes makes it difficult to advise strict follow-up for patients with small pox scars. One should be aware however that atypical changes and/or symptoms occurring in a small pox scar could potentially mean malignant transformation, and adequate diagnostic procedures such as local biopsy and/or primary/secondary wide local excision are the first appropriate steps.

## Conclusions

This report describes the first case of CLM formation in a small pox scar. Primary CLM can be a diagnostic challenge and adequate surgical excision is the appropriate treatment. Based on this one case, regular checks cannot be recommended. However, any change in the existing scar should be followed by a biopsy in order to exclude a sarcoma or make an early diagnosis.

## Consent

Written informed consent was obtained from the patient for publication of this report and accompanying images. A copy of this written consent is available for review by the Editor-in-Chief of this journal.

## Competing interests

The authors declare that they have no competing interest.

## Authors’ contributions

RP and HD drafted the manuscript. JLR drafted part of the manuscript, critically revised its final form, and provided Figures 2 and 3. RJG critically revised the final manuscript and was responsible for the primary care and outpatient controls. All authors read and approved the final manuscript.
